# The relationship between borderline personality disorder and self‐injurious/suicidal behaviors in adolescents and young adults: A protocol for systematic review and meta‐analysis

**DOI:** 10.1002/hsr2.2143

**Published:** 2024-06-11

**Authors:** Bhaskar Thakur, Mona Pathak, Chance Strenth, Kristin Wilmoth, Elizabeth Mayfield Arnold

**Affiliations:** ^1^ Department of Family and Community Medicine UT Southwestern Medical Center Dallas Texas USA; ^2^ Peter O'Donnell Jr. School of Public Health UT Southwestern Medical Center Dallas Texas USA; ^3^ Department of Pharmacotherapy UNT System College of Pharmacy Fort Worth Texas USA; ^4^ Departments of Psychiatry and Physical Medicine & Rehabilitation UT Southwestern Medical Center Dallas Texas USA; ^5^ Department of Psychiatry, College of Medicine University of Kentucky Lexington Kentucky USA

**Keywords:** borderline personality disorder, meta‐analysis, non‐suicidal self‐injury, suicide, systematic review

## Abstract

**Background & Aims:**

Borderline personality disorder (BPD) is a common psychiatric disorder associated with a high risk of suicide attempts, death by suicide, and non‐suicidal self‐injury (NSSI). A systematic and comprehensive understanding of the link between BPD and suicide and self‐injury in adolescents and young adults is crucial for effective public health prevention strategies. This protocol outlines our approach to summarize the evidence on the association between BPD diagnosis and self‐injurious/suicidal behaviors including death by suicide, nonfatal suicide attempts, NSSI, and self‐harm behavior through a systematic review and meta‐analysis.

**Methods:**

The protocol is registered (PROSPERO: CRD42022363329) and developed in accordance with the Preferred Reporting Items for Systematic Reviews and Meta‐Analyses Protocols (PRISMA‐P)‐2015 statement. We will conduct a comprehensive literature search using electronic databases including MEDLINE, EMBASE, SCOPUS, Web of Science, CINHAL, and PsycINFO. The review will include studies that meet the specific inclusion criteria and will be searched using multiple databases A meta‐analysis will be conducted using a fixed‐effects or random‐effects approach based on the level of heterogeneity. Subgroup analysis and meta‐regression will be performed if necessary.

**Conclusion:**

This study is unique, as it is the first of its kind to systematically review and analyze the existing literature on this topic. The results of this study will provide important evidence on the magnitude of this relationship overall and in different subgroups, which can be used to inform the development of effective prevention and treatment strategies.

## 1 BACKGROUND

Borderline personality disorder (BPD) is a common psychiatric disorder characterized by poor self‐image, feelings of emptiness, and great difficulty in self‐care practice, especially when staying alone.^1^, ^2^ As per the DSM‐5 criteria, a diagnosis of BPD is established when an individual demonstrates five out of nine specified symptoms and meets at least one criterion from at least three sectors.^3^ This disorder is associated with a high risk of death by suicide, nonfatal suicide attempts, non‐suicidal self‐injury (NSSI), diverse functional impairments, substantial treatment use, and high costs to society.^4^, ^5^, ^6^, ^7^ In a large‐scale nationally representative US survey, the point prevalence of BPD is 1.6%, with a lifetime prevalence of approximately 6%,^8^, ^9^ in community settings, the prevalence is roughly 1%, which increases to about 12% in outpatient psychiatric clinics and 22% in inpatient psychiatric clinics.^10^ Up to 10% of all BPD patients die by suicide.^11^ Recent research highlighted that among all types of personality disorders, BPD is the most significant factor associated with prospective suicide attempts (OR = 4.18), even after adjusting for potential covariates.^12^

Suicide is the 10th leading cause of death in the United States.^13^ In 2018, in the United States, more than 48,000 people died by suicide,^14^ and about 25 times that number attempted suicide.^15^ A nonfatal suicide attempt can lead to serious physical injury, long‐term effects on health, and brain damage. In addition to suicide attempts, NSSI has received increased attention in recent decades. A meta‐analysis reported the highest NSSI prevalence among adolescents (17.2%) compared to young adults at 13.4% and adults at only 5.5%.^16^ Evidence also shows that the lifetime prevalence rates of NSSI are 5.9% to 15.3% among adults with higher rates in females compared to males.^17^ However, males are at the greatest risk for suicide.^18^ Further people with a history of NSSI are more likely to attempt suicide compared to the general population.^18^

Some studies have shown associations between death by suicide, nonfatal suicide attempts, and NSSI between individuals with BPD and non‐BPD,^19^, ^20^, ^21^ however, the reported effect sizes in these studies are not consistent. A meta‐analysis study published more than a decade ago demonstrated that suicides among patients with BPD are more common than those in the general population^22^; however, they did not calculate an effect size to quantify the difference.

A detailed explanation of how the social determinants of health (SDoH), including gender, race, and ethnicity, can impact this relationship has not been closely examined. These variables are critical factors to examine given differences in BPD prevalence and self‐harm based. Female gender is linked with increased suicide attempts.^12^ Another study shows that nearly a third of youth suicides, most of whom were among males, can be diagnosed with BPD by psychological autopsy.^23^ Other studies of BPD patients who died by suicide also showed a preponderance of males.^24^ BPD prevalence is higher in females compared to males.^25^ Koenig and colleagues^26^ _ found negative affective states to predict incidents of NSSI within the next hour among female adolescents which also aligns with other evidence.^27^, ^28^ Non‐Hispanic American Indian and Alaska Native (AI/AN) people and non‐Hispanic White people have the highest age‐adjusted suicide rates: 28.1 per 100,000 and 17.4 per 100,000, respectively.^29^ Research suggests that Hispanic (primarily Mexican American) adolescents report higher rates of suicidal ideation than their White peers,^30^, ^31^, ^32^ followed by a sentence “However, a detailed explanation of how the social determinants of health, including gender, race and ethnicity, can impact this relationship has not been fully explored among those diagnosed with BPD.”

This systematic review aims to address critical gaps in understanding the association between BPD and suicide‐related outcomes, including death by suicide, nonfatal suicide attempts, and NSSI, particularly among adolescents and young adults. Given the prevalence and severity of BPD, along with its substantial impact on individuals and society, clarifying these associations is important. It is noteworthy that, to date, no meta‐analysis has been conducted to generate evidence separately for adolescent and adulthood groups, nor has exploration into variables such as gender, race, and ethnicity been thoroughly undertaken. With suicide being a significant public health concern, especially among vulnerable populations, such as those with BPD, comprehensive research is needed to inform targeted interventions. By exploring gender, race, and ethnic disparities in these associations, as well as differences between adolescents and adults, this study seeks to provide detailed insights that can guide adapted interventions and improve overall mental health outcomes.

The systematic review and meta‐analysis aim to address the following research questions using the PECO framework^33^: Population (P): Adolescents and young adults; Exposure (E): BPD); Comparator (C): Non‐BPD); and Outcomes (O): death by suicide, nonfatal suicide attempts, NSSI, and self‐harm behavior.

The PECO‐based main objectives of this systematic review and meta‐analysis are as follows: 1. To examine the association of BPD with death by suicide, nonfatal suicide attempts, NSSI, and self‐harm behavior in adolescents and young adults; 2. To examine the gender and ethnic disparities in the association of death by suicide, nonfatal suicide attempts, and NSSI between BPD and non‐BPD in adolescents and young adults; and 3. To identify adolescent and adulthood disparities in the association of death by suicide, nonfatal suicide attempts, and NSSI between BPD and non‐BPD.

## 2 METHODS

This systematic review and meta‐analysis protocol was developed following the Preferred Reporting Items for Systematic Reviews and Meta‐analyses Protocols (PRISMA‐P)‐2015 statement,^34^ The protocol for this systematic review has been registered in the international prospective register of systematic reviews PROSPERO network (PROSPERO: CRD42022363329). Since this is a protocol for systematic review and meta‐analysis and we will not be engaging in research activities with any study participants, ethical approval and informed consent are not required.

### 2.1 Inclusion/exclusion criteria

#### 2.1.1 Types of studies

Observational studies (analytical cross‐sectional, case‐control, and longitudinal cohort studies) written in English will be eligible for inclusion. All the studies must have a control/comparison group. Case reports, case series, review articles, and recommendations/guidelines will be also excluded. The review inclusion criteria are specified according to PECO guidelines.

#### 2.1.2 Populations

The studies involving common age criteria for adolescents are typically 13–18 years, and for young adults, 19–44 years. However, it is presumed that the age criteria for these two populations may vary slightly from one study to another (across various sources) especially within the young adult population. Therefore, there will be no restrictions on the defined age criteria for young adults as reported in the articles that will be reviewed. Further, no restrictions will be applied to gender, ethnicity, race, other socioeconomic and demographic characteristics, or comorbid illnesses.

#### 2.1.3 Exposures

Studies must indicate the diagnosis of BPD according to the different version of Diagnostic and Statistical Manual of Mental Disorders or emotionally unstable personality disorder according to the International Classification of Diseases (ICD‐9 or ICD‐10) system.

#### 2.1.4 Comparators

Studies must include a control group for self‐injurious behavior in adolescents/young adults without BPD.

#### 2.1.5 Outcomes measures

Studies reporting suicide and harm‐related information will be eligible. This includes death by suicide, which denotes the deliberate act of self‐inflicted death; nonfatal suicide attempts, referring to behaviors with intent to die but do not result in death; NSSI, representing deliberate self‐harm devoid of suicidal intent; and self‐harm behavior, refereeing as any type of self‐injurious behavior, including suicidal attempt or NSSI. Each of these recorded outcomes will be precisely assessed and categorized according to the original study authors' documentation. In addition, a composite outcome is based on all the reported individual outcomes, that is, death by suicide, nonfatal suicide attempt, or NSSI will be created.

### 2.2 Exclusion criteria

In the case of encountering any linked/duplicate studies (representing the same cohort or study center with similar years), we will keep the more informative and powered study and will exclude the others, if any. Subjects diagnosed with personality disorders other than BPD will be excluded. Any screened full‐text studies that have not estimated the relevant effect sizes or other required quantitative outcome measures will be excluded. Studies published in languages other than English language will also be excluded. There will be no restrictions on geographical boundaries.

### 2.3 Information sources and search strategy

#### 2.3.1 Electronic search

To identify relevant studies, a comprehensive literature review will be performed from inception up to November 30, 2023 using a search of electronic databases including MEDLINE (via PubMed), EMBASE, SCOPUS, Web of Science, CINHAL, and PsycINFO.

After analyzing the key studies and considering expert recommendations, the following keywords are identified for conducting the search: personality disorder, borderline personality disorder, borderline symptoms, suicide attempt, suicidal attempt, suicide, suicidal death, suicide death, death by suicide, self‐injury, self‐harm, suicidal ideation, suicide idea, suicidal idea, suicidal behaviors.

#### 2.3.2 Google scholar

The search will include up to 10 pages to identify all relevant literature for this meta‐analysis.

#### 2.3.3 Other sources (reference lists)

The reference lists of earlier meta‐analyses,^22^ related review articles, and all screened eligible studies will be searched for additional eligible articles.

#### 2.3.4 Search strategy

The search terms relating to BPD (*personality disorder* OR *borderline personality disorder* OR *borderline symptoms* with MesH terms) will be combined with terms relating to suicidal outcomes (*suicide attempt* OR *suicidal attempt* OR *suicide* OR *suicidal death* OR *suicide death* OR *death by suicide* OR *self‐injury* OR *self‐harm* OR *suicidal ideation* OR *suicide idea* OR *suicidal idea* OR *suicidal behaviors* with MesH terms) and with the target populations (*adolescent* OR *young adult* with MesH terms). Equivalent Index Terms will be utilized in PsycINFO. Detailed search strategies are provided in Table 1.

**Table 1:** Search strategies for identifying relevant studies.

**PubMed**	(“Borderline Personality Disorder”[Mesh] OR “borderline personality disorder”[Title/Abstract]) AND (“Suicide”[Mesh] OR “Suicide, Attempted”[Mesh] OR “Suicide, Completed”[Mesh] OR “Self‐Injurious Behavior”[Mesh] OR “suicid*“[Title/Abstract] OR “self mutilation”[MeSH Terms] OR “self injur*“[Title/Abstract] OR “self poison*“[Title/Abstract] OR “suicidal ideation”[MeSH Terms]) AND (“Young Adult”[Mesh] OR “Adolescent”[Mesh]) NOT(booksdocs[Filter] OR meta‐analysis[Filter] OR review[Filter] OR systematicreview[Filter] OR randomizedcontrolledtrial[Filter])
**SCOPUS**	(TITLE‐ABS‐KEY (“borderline personality disorder”) AND TITLE‐ABS‐KEY (suicid* OR (self PRE/0 harm) OR (self AND pre/0injur) OR (self PRE/0 mutil*) OR “suicidal ideation”) AND TITLE‐ABS‐KEY (adolescen* OR (young AND adult) OR junior OR juvenile OR minor OR pubescent OR youn*))
**Web of science**	TS = (“borderline personality disorder”) AND TS = (suicid* OR self?harm OR self?injur* OR self?mutil* OR suicid?idea*) AND TS = (adolescen* OR young?adult OR junior OR juvenile OR minor OR pubescent OR youn*)
**CINHAL**	(TX “borderline personality disorder” OR MH (“borderline personality disorder”)) AND (MW (suicid* OR (self‐harm) OR (self‐injur*) OR (self mutil*) OR (suicid* idea*)) OR TX(suicid* OR (self‐harm) OR (self‐injur*) OR (self mutil*) OR (suicid* idea*))) Limiters ‐ Human; Age Groups: Adolescent: 13‐18 years, Adult: 19‐44 years
**PsychInfo via APA psycNet**	(Abstract: borderline personality disorder) AND (Abstract: suicid* OR (Abstract: self‐harm) OR (Abstract: self injur*) OR (Abstract: self mutil*) OR (Abstract: suicid* idea*)) *Filter Age Group: Adolescence (13*–*17 yrs) OR Young Adulthood (18*–*29 yrs)*
**EMBASE via Ovid (UTSW library)**	*Multi‐Field Search* exp borderline state/and (exp suicidal ideation/or exp suicide attempt/or exp suicide/or exp suicidal ideation/or exp automutilation/) and (exp young adult/or exp adolescent/) and exp human/

### 2.4 Study records

#### 2.4.1 Data management

All references will be imported into Endnote software (Thompson Reuters). Identified duplicate documents will be removed, and the rest of the references assessed for eligibility will be sorted alphabetically according to the first author's last names.

#### 2.4.2 Selection process

**Figure 1:**
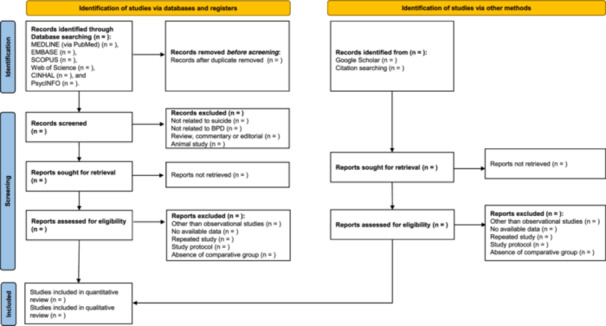
Preferred Reporting Items for Systematic Reviews and Meta‐analyses Protocols (PRISMA‐P) 2015 flow diagram for systematic reviews which shows the study selection process.

Two authors (Bhaskar Thakur and Mona Pathak) will independently screen the titles and abstracts of the studies identified by the search strategy after excluding duplicate documents. The screened studies identified by either of the two authors will be reassessed by reading the full text independently by both authors. In case of disagreement, the opinions of a third author (Kristin Wilmoth) and a senior author (Elizabeth Mayfield Arnold) will be considered. According to PRISMA‐P 2015 guidelines^34^ the flow diagram demonstrates the study selection process (Figure 1).

#### 2.4.3 Data collection process

An author (Bhaskar Thakur) will complete the data extraction from selected studies A second author (Chance Strenth) will check the accuracy and consistency of all the extracted information and will make the appropriate suggestions and clarification if needed. A third author (Mona Pathak) will be requested to resolve any disagreements regarding data extraction.

#### 2.4.4 Data items

Data will be collected on the following items: name of the first author, year of publication, country of study, study design, setting, total number of subjects/participants, number of participants in the exposed/case group, number of participants in the unexposed/control group, type of control/unexposed group, assessment tool for personality disorder, participant demographics (gender, age, marital status, ethnicity, and race) and cultural background (relationship, language, religious/rituals belief/affiliation, religion), physical activity, BMI/obesity status, locality (rural/urban), educational status, psychiatric history in the family, parental educational status, information on comorbidities, information on childhood adversity, suicide attempt (yes/no), method of suicide attempt, death by suicide (yes/no), NSSI (yes/no), and NSSI method.

### 2.5 Outcomes and prioritization

Death by suicide, nonfatal suicide attempts, and NSSI will be considered eligible for inclusion. In addition, a composite outcome based on all the reported outcomes, that is, death by suicide or nonfatal suicide attempt, and NSSI will be created.

### 2.6 Quality and risk of bias assessment

The methodological quality of observational studies will be performed differently for case‐control studies, cohort studies, and cross‐sectional studies using the Newcastle Ottawa Scale (NOS).^35^ All the included studies will obtain a score based on the scoring composition of three risks of bias domains in the coding manual for a specific study design, which will range from 0 to 9 stars: selection (will be rated on a 0–4 stars), comparability (BPD and non‐BPD group) (will be rated on a 0–2 star), and assessment of exposure (will be rated on a 0–3 star). The overall score will be converted into good, fair, or poor quality according to the Agency for Healthcare Research and Quality (AHRQ) standards. The methodological quality will be evaluated independently by two authors (Bhaskar Thakur and Chance Strenth). A third author (Mona Pathak) and a senior author (Elizabeth Mayfield Arnold) will be consulted to resolve the lack of consensus.

### 2.7 Statistical analysis

#### 2.7.1 Process before quantitative synthesis

An ad hoc table will be designed to report the study's key characteristics and any relevant information related to the goal of this review. Reviewers will determine the possibility of meta‐analysis after data extraction. If the quantitative data sought are sufficient for meta‐analysis, all the extracted data on effect sizes for the outcomes from the primarily included studies will be converted into a meaningful effect size scale which will be an odds ratio (to keep the effect sizes consistent) if already not available. The data will then be combined using the statistical method, and the pooled effect with a 95% confidence interval (CI) will be reported.

#### 2.7.2 Method of quantitative synthesis

A fixed‐effects meta‐analysis will be performed if there is no evidence of heterogeneity. The included studies may be heterogeneous due to methodological and sample variations and variation in the outcome measures according to geography, diverse demographic, and cultural backgrounds more than expected by chance alone. In such a case, the DerSimonian and Laird method of random effects with the inverse‐variance model^36^, ^37^ will be used to synthesize study‐level effect sizes i.e. odds ratios with 95% CI. The reported study‐level effect sizes measured using different indices will be converted to a common index before combining these estimates in a meta‐analysis. Heterogeneity will be assessed by visual inspection of the forest plot by performing the χ2 test (assessing the p‐value) and by calculating the I^2^ statistic, which describes the percentage of observed heterogeneity that would not be expected by chance. We will consider substantial heterogeneity if either the p‐value is smaller than 0.10, or the I‐square exceeds 50%.^38^, ^39^ Usually, the I^2^ value is considered small if 0 ≤ I^2^ ≤ 25%, medium if 25% < I^2^ ≤ 50%, and large if I^2^ > 50%. To explore the sources of heterogeneity in meta‐analytic data, meta‐regression analysis will be used.^40^ This analysis allows users to investigate the relationship between study‐level characteristics and effect sizes. The Computed *P* values will also be supported. Publication bias will be visualized using a funnel plot asymmetry^41^ and will be tested using Egger test.

All statistical tests which will be employed in this study will be conducted as two‐sided tests. For P values less than 0.001, we will report as *“p* < 0.001”; for P values ranging from 0.001 to 0.01, we will present the value rounded to the nearest thousandth; for P values equal to or greater than 0.01, we will provide the value rounded to the nearest hundredth; and for P values exceeding 0.99, we will indicate as *“p* > 0.99”. All the analysis will be performed on Stata 18.0.^42^ To perform the meta‐analysis of aggregate data, we will use a Stata package called “*metan*” with an option “random” to specify the DerSimonian‐Laird random‐effects model. Using the “*metareg*” package, we will perform the meta‐regression analysis. We will further use the package “*metabias*” to perform the Egger test and funnel‐plot asymmetry in meta‐analysis for publication bias assessment. Our reporting of statistics also complies with the previous recommendation.^37^, ^43^ The strength of our cumulative evidence studies will be evaluated using the Oxford Centre for Evidence‐Based Medicine.^44^

#### 2.7.3 Sensitivity analysis

A sensitivity analysis will be performed by excluding poor‐quality studies from the analysis. Additionally, the studies will be analyzed separately for each study design.

#### 2.7.4 Subgroup analysis

Subgrouping in the analysis will be performed based on gender, race, and ethnicity (Hispanic vs. non‐Hispanic) with the availability of a suitable number of studies at each subgroup level.

#### 2.7.5 Unfeasible quantitative synthesis

If there is substantial heterogeneity (in terms of common characteristics) among the studies, and a meta‐analysis is not possible, only a systematic review with descriptive analysis will be conducted.

### 2.8 Dissemination

The results will be disseminated through national or international presentations, and the publication of the manuscript in a peer‐reviewed journal.

## 3 DISCUSSION

To date, no review has comprehensively assessed the rate of death by suicide, nonfatal suicide attempts, and NSSI among adolescents and young adults with or without borderline personality disorder. Pompili et al. conducted a 2005 meta‐analysis, which showed that death by suicide among patients with a borderline personality disorder is more frequent than in the general population.^22^ However, a more recent update of the literature that includes information on effect sizes is needed. Although BPD and suicidality have been a topic of other protocols,^45^ there has not yet been a review specifically targeting younger individuals and SDoHs.

To our knowledge, our proposed systematic review and meta‐analysis will be the first to report how deaths by suicide, nonfatal suicide attempts, and NSSI are related to adolescents and young adults with BPD. Furthermore, this review will describe the gender and ethnic disparities in such associations if a reasonable number of studies are available.

This review could be restricted by the characteristics of the included studies and their methodological quality. We will conduct and report our review using existing recommendations^43^, ^44^ along with a methodological quality assessment. This protocol will provide an overview of step‐by‐step guidelines, following the PRISMA 2020 statement,^46^ to conduct the planning and design of a systematic review and meta‐analysis of observational studies,^47^ particularly those related to BPD and suicide.

This study will have broad representativeness by including a more detailed and comprehensive view on not only the association of either composite outcome measure or each of the outcome measures (i.e., deaths by suicide, nonfatal suicide attempts, and NSSI) among individuals with BPD,, but also examining the gender and ethnic disparities in such associations. The protocol provides an intelligible and structured strategy to extract relevant information and summarizes information regarding hypothesized associations. The findings from this systematic review and meta‐analysis will be of benefit to readers, practitioners, researchers, and policymakers engaged in the management of personality disorders, suicidality, and self‐harm. However, there are some inherent limitations to conducting a systematic review and meta‐analysis. Studies published only in the English language will be included. There could have been publication bias and poor method quality. However, some strategies (explained in the methodology section) will be adopted to assess such biases. Furthermore, available guidelines such as MOOSE,^47^ and PRISMA 2020 statement^46^ will be followed.

## AUTHOR CONTRIBUTIONS

The study concept was developed using the Bhaskar Thakur and Elizabeth Mayfield Arnold. The search strategy was developed by all authors, and the search will be performed using Bhaskar Thakur and Mona Pathak. The manuscript was drafted by Bhaskar Thakur and critically revised by Mona Pathak, Chance Strenth, Elizabeth Mayfield Arnold, and Kristin Wilmoth. Study selection will be carried out primarily by Bhaskar Thakur, Mona Pathak, Kristin Wilmoth, and Elizabeth Mayfield Arnold in case of disagreements. Data extraction was performed using the Bhaskar Thakur and Chance Strenth. Methodological quality assessments will be performed primarily by Bhaskar Thakur and Chance Strenth and by Mona Pathak in case of disagreements. All authors have read and approved the final version of the manuscript. All the authors will have full access to all the data for this systematic review and will take complete responsibility for the integrity of the data and the accuracy of the data analysis.

## CONFLICT OF INTEREST STATEMENT

The authors declare no conflict of interest.

## Data Availability

Data sharing is not relevant to this manuscript as it is protocol paper with no data reported.
